# The Tissue Selective Estrogen Complex: A Promising New Menopausal Therapy

**DOI:** 10.3390/ph5090899

**Published:** 2012-09-04

**Authors:** Barry S. Komm, Sebastian Mirkin

**Affiliations:** Women’s Health, Pfizer Inc., 500 Arcola Rd., Collegeville, PA 19426, USA; Email: sebastian.mirkin@pfizer.com

**Keywords:** hormone therapy, tissue selective estrogen complex (TSEC), bazedoxifene, conjugated estrogens, menopause

## Abstract

Menopause is associated with health concerns including vasomotor symptoms, vulvar/vaginal atrophy (VVA), and osteoporosis. Estrogen therapy or combined estrogen-progestin therapy (EPT) are primary treatment options for menopausal symptom relief and osteoporosis prevention. Because EPT has been associated with some safety/tolerability concerns relating to undesirable effects of estrogen and progestin, alternative options are needed. The tissue selective estrogen complex (TSEC) is a novel class of agents pairing a selective estrogen receptor modulator (SERM) with 1 or more estrogens. The TSEC combines the established efficacy of estrogens on menopausal symptoms and bone with the protective effects of a SERM on the reproductive tract. The pairing of bazedoxifene (BZA) with conjugated estrogens (CE) has been evaluated in a series of phase 3 clinical trials. BZA 20 mg/CE 0.45 mg and BZA 20 mg/CE 0.625 mg have shown efficacy in reducing the frequency and severity of hot flushes, relieving VVA symptoms, and maintaining bone mass while protecting the endometrium and breast. These BZA/CE doses have been associated with a favorable safety/tolerability profile, with higher rates of cumulative amenorrhea and lower incidences of breast pain than those reported for EPT. Thus, BZA/CE may be a promising alternative to conventional EPT for treating non-hysterectomized, postmenopausal women.

## 1. Introduction

The menopausal transition is associated with a decline in ovarian production of endogenous estrogens [1,2], leading to a state of estrogen deficiency that can result in some bothersome symptoms and the loss of bone mass. Vasomotor symptoms (VMS) are experienced by 60% to 90% of women and can interfere with sleep and daily activities [3]. Vulvar/vaginal atrophy (VVA) can result in uncomfortable symptoms such as vaginal dryness [4]; it has been reported that the percentage of women with vaginal dryness increases from 25% in the first year postmenopause to 47% in the third year postmenopause [5]. Moreover, the loss of bone mass associated with postmenopausal estrogen deficiency can increase the risk of osteoporosis and bone fractures [6,7]. Overall, menopausal symptoms can adversely affect the quality of life for women and impose a significant economic burden in the form of decreased productivity and increased health care costs [4,8]. Hormone therapy (HT) is the only treatment option for both menopausal symptoms and osteoporosis prevention; other options are available for either treatment of menopausal symptoms or osteoporosis, but each of these options carries a unique risk/benefit profile and thus may not be appropriate for all women. The objective of this article is to review HT and other therapies for the treatment of menopausal symptoms and prevention of osteoporosis, and to describe the tissue selective estrogen complex (TSEC), a promising new class of therapy for postmenopausal women.

## 2. Hormone Therapy

### 2.1. Types of HT

HT, in the form of estrogen therapy (ET; for hysterectomized women) or combined estrogen-progestin therapy (EPT; for non-hysterectomized women), is the established treatment for postmenopausal women with moderate-to-severe VMS and VVA. ET and EPT are also approved for the prevention of postmenopausal osteoporosis [9,10]. Estrogens have broad and varied pharmacologic effects in different tissues, some of which are desired and others undesired. Unopposed estrogens are associated with an increased risk of endometrial carcinoma [11,12,13,14], and so progestins are added in EPT for women with an intact uterus in order to protect the endometrium [9,15]. Progestins also have varied pharmacologic effects, which are complex, both desirable and undesirable, and may also depend on estrogen. There are multiple formulations available for both ET and EPT; in the United States, oral conjugated estrogens (CE) is the most commonly prescribed ET and medroxyprogesterone acetate (MPA) is the most commonly used progestin for EPT [16].

### 2.2. Efficacy of HT on Menopausal Symptoms

HT is primarily indicated for the treatment of moderate-to-severe VMS [9], and the efficacy of HT in relieving VMS has been established in numerous clinical trials in postmenopausal women [16,17,18,19,20,21,22]. A systematic review of randomized, double-blind, and placebo (PBO)-controlled studies of oral HT (including both ET and EPT) showed a 75% reduction in the frequency of hot flushes (95% confidence interval [CI], 64.3–82.3) for HT compared with PBO [17]. HT was also associated with a significant reduction in the severity of hot flushes (odds ratio [OR], 0.13; 95% CI, 0.07–0.23) compared with PBO [17].

HT has been shown to be effective in improving VVA symptoms in postmenopausal women, with ET and EPT both showing improvements in measures of VVA, including vaginal maturation index and vaginal dryness, compared with PBO [16,21,23]. A meta-analysis reported that local application of estrogens is as effective as systemic estrogens for the treatment of VVA symptoms [23]; local estrogens are recommended for women who are seeking treatment for VVA symptoms only [9,24]. As noted by the North American Menopause Society, hepatic metabolism that occurs with oral administration may result in the requirement for higher doses compared with vaginal estrogen delivery to achieve local concentrations of estrogens high enough to provide symptomatic relief and reversal of atrophic changes [24]. For example, local estrogen delivery using a low-dose (0.3 mg) CE cream had little to no impact on serum estrogen levels compared with oral administration at the same dose [25], but significantly improved participant-reported most bothersome symptom of VVA compared with PBO and significantly decreased dyspareunia compared with PBO at 12 weeks [26].

### 2.3. Effects of HT on Bone

HT has demonstrated efficacy in preventing bone loss and is approved for the prevention of postmenopausal osteoporosis [9,10]. In the Women’s Health Initiative (WHI) trial, ET with conjugated equine estrogens (CEE) (*p* < 0.0001) [27] and EPT with CEE/MPA (*p* < 0.001) [28] significantly improved total hip bone mineral density (BMD) compared with PBO over 6 and 3 years, respectively. These findings are consistent with those from the previous Women’s Health, Osteoporosis, Progestin, Estrogen (HOPE) trial showing a significant improvement in spine and hip BMD over 2 years for women who received CEE or CEE/MPA compared with those who received PBO (*p* < 0.001) [29]. More importantly, both CEE and CEE/MPA also showed significant reductions in total fracture risk in the WHI trial (hazard ratio [HR] of 0.71 and 95% CI of 0.64–0.80 for CEE; HR of 0.76 and 95% CI of 0.69–0.83 for CEE/MPA) compared with PBO [27,28]. Similarly, meta-analyses of randomized trials have also shown reduced risk of fractures and improvement in BMD for HT [30,31]. In a meta-analysis of 22 trials in which women received at least 12 months of HT, there was a 27% reduction in nonvertebral fracture risk favoring HT (relative risk [RR], 0.73; 95% CI, 0.56–0.94; *p* = 0.02) [30]. Interestingly, nonvertebral fracture risk reduction was greater in women randomized to HT with mean age <60 years (RR, 0.67; 95% CI, 0.46–0.98; *p* = 0.03) than in those with mean age ≥60 years (RR, 0.88; 95% CI, 0.71–1.08; *p* = 0.22). In another meta-analysis that included 57 studies of postmenopausal women randomized to HT or control (PBO or calcium/vitamin D) for at least 1 year, there was a nonsignificant trend toward a reduced incidence of vertebral (RR, 0.66; 95% CI, 0.41–1.07; 5 trials) and nonvertebral fractures (RR, 0.87; 95% CI, 0.71–1.08; 6 trials) with HT and consistent, favorable effects on BMD [31]. Taken together, these data suggest that HT may be appropriate for a younger population that may require long-term osteoporosis prevention [9].

### 2.4. Effects of HT on Sleep and Quality of Life

HT has been reported to improve sleep parameters in several studies. In the WHI trial, both CEE and CEE/MPA provided small but statistically significant improvements in sleep disturbance compared with PBO at 1 year (*p* < 0.001) [32,33]. These results are consistent with findings from previous studies showing improvements in sleep parameters for ET and EPT [34,35]. The relief of VMS with ET was shown to be the most important predictive factor for a positive treatment effect on sleep parameters [34]. In contrast, studies examining the effects of HT on measures of quality of life have shown mixed results. One study showed a significant improvement in the Menopause-Specific Quality of Life (MENQOL) summary score for EPT (estradiol/norgestimate) compared with PBO (*p* < 0.001) [36] and the Heart and Estrogen/Progestin Replacement Study (HERS) trial showed improved mental health and depressive symptoms for EPT (CEE/MPA) compared with PBO (*p* = 0.04 and *p* = 0.01, respectively) for women experiencing hot flushes at baseline, but not for those without hot flushes at baseline [37]. Other studies, including the WHI trial, have shown no clinically meaningful improvements in measures of quality of life for ET (estradiol) and EPT (CEE/MPA) [32,33,38].

### 2.5. Safety and Tolerability of HT

Findings from the WHI trial have raised some safety concerns with HT and, in particular, with CEE/MPA [39].

#### 2.5.1. Estrogen Therapy

CEE alone showed no overall increase in the incidence of coronary heart disease (CHD; HR, 0.91; 95% CI, 0.75–1.12) in the WHI trial, although a trend toward increased risk of peripheral arterial events (categorized as carotid artery disease, abdominal aortic aneurysm, or lower extremity arterial disease) was observed (HR, 1.32; 95% CI, 0.99–1.77) [40,41]. Risk of CHD associated with CEE therapy was somewhat increased in women aged 70 to 79 years (HR, 1.13; 95% CI, 0.82–1.54) but there was reduced risk of CHD in women aged 50 to 59 years (HR, 0.63; 95% CI, 0.36–1.09) (Table 1) [42]. In an ancillary substudy of women aged 50 to 59 years participating in the WHI trial, calcified-plaque burden in the coronary arteries, as measured by change in coronary artery calcium scores, was significantly lower with CEE *vs*. PBO (*p* = 0.02) [43].

An increased risk of stroke and venous thromboembolic events (VTEs) was observed with CEE during the WHI trial [41], and these risks were generally greater in older women compared with younger women [44,45]. The overall HR for stroke was 1.37 (95% CI, 1.09–1.73; *p* = 0.008) [44]; however, CEE alone was associated with a decreased risk of stroke in a subgroup of younger women aged 50 to 59 years [42]. For VTEs, there was a 32% increased risk with CEE overall (HR, 1.32; 95% CI, 0.99–1.75); risk was lower in women aged 50 to 59 years at baseline (HR, 1.37; 95% CI, 0.70–2.68) than in women aged 70 to 79 years at baseline (HR, 3.77; 95% CI, 2.07–6.89) (Table 1) [45].

HT, particularly EPT, has also been associated with some breast-related safety concerns, which are discussed below. No increase in the risk of invasive breast cancer was initially observed for CEE compared with PBO in the WHI trial (HR, 0.80; 95% CI, 0.62–1.04) [46]. At a mean follow-up of 10.7 years, CEE showed a significantly decreased risk of breast cancer compared with PBO (HR, 0.77; 95% CI, 0.62–0.95; *p* = 0.02) and no increased or decreased risk of total mortality (HR, 1.02; 95% CI, 0.91–1.15) [47].

**Table 1:** Summary of key cardiovascular outcomes with hormone therapy analyzed by age group.

HR (95% CI)	Age group at randomization		
	50 to 59 years	60 to 69 years	70 to 79 years
CHD [42]			
CEE alone	0.63 (0.36–1.09)	0.94 (0.71–1.24)	1.13 (0.82–1.54)
CEE/MPA	1.29 (0.79–2.12)	1.03 (0.74–1.43)	1.48 (1.04–2.11)
Stroke [42]			
CEE alone	0.89 (0.47–1.69)	1.62 (1.15–2.27)	1.21 (0.84–1.75)
CEE/MPA	1.41 (0.75–2.65)	1.37 (0.95–1.97)	1.21 (0.82–1.78)
VTE [45,48]			
CEE alone	1.37 (0.70–2.68)	2.82 (1.59–5.01)	3.77 (2.07–6.89)
CEE/MPA	2.27 (1.19–4.33)	4.28 (2.38–7.72)	7.46 (4.32–14.38)

HR, hazard ratio; CI, confidence interval; CHD, coronary heart disease; CEE, conjugated equine estrogens; MPA, medroxyprogesterone acetate; VTE, venous thromboembolism.

#### 2.5.2. Estrogen-Progestin Therapy

In contrast to CEE alone, CEE/MPA was associated with a 29% increase in the incidence of CHD (HR, 1.29; 95% CI, 1.02–1.63; *p* < 0.05) in the WHI trial [39]. Further analyses have shown that the risk of CHD correlates with the timing of CEE/MPA initiation, with increased risk of CHD observed for women who were ≥20 years past menopause (HR, 1.66; 95% CI, 1.14–2.41) while younger women <10 years past the onset of menopause at HT initiation showed a risk of CHD comparable to PBO (HR, 0.88; 95% CI, 0.54–1.43); this trend toward decreased risk with decreasing time since menopause was statistically significant (*p* = 0.05) [42]. When analyzed by age at initiation of CEE/MPA, CHD risk remained consistent across age groups (Table 1) [42].

Similar to CEE alone, CEE/MPA was also associated with an increased risk of stroke and VTEs during the WHI trial [39], with risks generally greater in older *vs*. younger women [48,49]. The overall HR for stroke was 1.31 (95% CI, 1.02–1.68) for CEE/MPA [49], with an increased risk in the subgroup of younger women aged 50 to 59 years (Table 1) [42]. When stroke risk data from the CEE and CEE/MPA studies were combined, there was no significant increase in the risk of stroke in this subgroup (HR, 1.13; 95% CI, 0.73–1.76). For VTEs, greater risk was observed with CEE/MPA, which showed a 2-fold increase (HR, 2.06; 95% CI, 1.57–2.70), compared with the 32% increase with CEE described above [45,48]. The HR for VTEs in women aged 50 to 59 years at baseline was lower (2.27 [95% CI, 1.19–4.33]) compared with that for women aged 70 to 79 years at baseline (7.46 [95% CI, 4.32–14.38] for EPT) (Table 1) [48]. For women aged 50 to 59 years at randomization, the absolute excess risk of VTEs with either CEE or CEE/MPA is considered rare [9].

As mentioned above, EPT has been associated with some breast-related safety concerns. HT has been shown to increase mammographic breast density in postmenopausal women, with EPT having a greater effect on breast density compared with ET [50,51,52,53]. High mammographic breast density has been shown to be a risk factor for breast cancer [54], as confirmed in a recent systemic meta-analysis [55]; however, this relationship is not well understood. High breast density may also decrease the sensitivity of mammograms for detecting breast abnormalities [56]. In the WHI trial, CEE/MPA was associated with a 26% increase in the incidence of invasive breast cancer (HR, 1.26; 95% CI, 1.00–1.59) [39,57] and with increased breast cancer mortality (HR, 1.96; 95% CI, 1.00–4.04) [58] compared with PBO. The increase in absolute risk of invasive breast cancer for CEE/MPA compared with PBO was 8 cases per 10,000 women-years, which is considered rare [9].

EPT has also been associated with some tolerability issues including increased incidences of breast pain and irregular vaginal bleeding [35,59,60]. In the WHI trial, women receiving CEE/MPA who were asymptomatic at baseline were significantly more likely to develop breast tenderness than women receiving PBO (*p* < 0.001) [60]; additional analyses have also linked new-onset breast tenderness after initiating CEE/MPA (but not CEE) to an increased risk of breast cancer (HR, 1.33; 95% CI, 1.02–1.72; *p* = 0.03) [61]. Vaginal bleeding was reported by 51% of women on CEE/MPA and only 5% of women on PBO at 6 months during the WHI trial [60]. Likewise, in the Heart and Estrogen/Progestin Replacement Study, women receiving CEE/MPA were more likely to experience bleeding and breast symptoms than those receiving PBO [35]. In the Postmenopausal Estrogen/Progestin Interventions Trial, breast tenderness was associated with EPT but not ET [18]. In a retrospective study to determine compliance with HT among 821 women attending a menopause clinic, the most common reason women discontinued therapy was irregular vaginal bleeding (23%) [59].

The North American Menopause Society currently recommends that HT be used at the lowest effective dose to treat postmenopausal women for whom the benefits of treatment outweigh the risks [9]. Overall, the benefit-to-risk ratio for HT, particularly ET, is favorable for younger women initiating therapy close to the onset of menopause than for older women who are many years postmenopause [9]. In general, when a specific population is carefully chosen, ET is not associated with an increased risk of many of these safety concerns. Assessment of the benefits and risks of HT for each individual woman is therefore important for determining whether HT may be appropriate.

Review of the clinical data above suggests that many of the concerns associated with HT appear to be related to the progestin component, as CHD risk, increased breast density, breast cancer risk, breast pain, and irregular vaginal bleeding are more often associated with EPT (particularly CEE/MPA) than with ET (*i.e*., CEE alone). These effects are broadly consistent with the known pharmacologic and physiologic effects of progestins. For example, in normal menstrual cycles in pre-menopausal women, progesterone acts to protect the endometrium from the proliferative effect of estrogens, but also promotes menstrual bleeding [62]. Moreover, there are differential effects with different types of progestins. In a French study that compared the association between different HTs and breast cancer risk, there was no association between route of estrogen administration and risk; however, risk of breast cancer with estrogen-progesterone compared with women who had never used HT (RR, 1.00; 95% CI, 0.83–1.22) was similar to the risk with estrogen alone (RR, 1.29; 95% CI, 1.02–1.65) and significantly lower than the risk with estrogen-other progestogens, including MPA (RR, 1.69; 95% CI, 1.50–1.91; *p* for homogeneity <0.001) [63]. Therefore, alternatives to progestin are needed that will protect the endometrium while avoiding other progestin-associated effects and preserving the desired effects of estrogens in postmenopausal women.

## 3. Other Therapies for Menopausal Symptoms or Postmenopausal Osteoporosis

For women who cannot or do not wish to take HT, other options include therapies that are targeted specifically to either menopausal symptoms or the treatment and/or prevention of osteoporosis. Some nonhormonal therapies have been prescribed off-label for the treatment of VMS. Agents such as selective serotonin reuptake inhibitors, serotonin norepinephrine reuptake inhibitors, clonidine, and gabapentin have shown some efficacy in reducing VMS, but are generally not as effective as HT [64]. Nonprescription remedies such as soy-based isoflavones have also been reported to provide some relief of menopausal symptoms, although clinical trials have shown mixed efficacy results [65,66]. For the treatment and/or prevention of postmenopausal osteoporosis, pharmacologic options include bisphosphonates, salmon calcitonin, parathyroid hormone, strontium ranelate (outside of the U.S.), denosumab, and selective estrogen receptor modulators (SERMs) [10,67]. Some of these therapies are associated with adverse effects with long-term treatment, are indicated for a restricted period of treatment, or are recommended only for women who are ≥5 years past the onset of menopause [10], thus limiting the treatment options for younger women at risk for osteoporosis who expect to be on therapy for many years. There is therefore an ongoing need for new treatment options for postmenopausal women seeking a comprehensive therapy for menopausal symptoms and prevention/treatment of osteoporosis that has a favorable long-term safety and tolerability profile.

### 3.1. The Tissue Selective Estrogen Complex

A novel menopausal therapy in clinical development is the tissue selective estrogen complex (TSEC), which partners a SERM with one or more estrogens [68]. SERMs can have either agonist or antagonist effects on the estrogen receptor (ER) depending on tissue type [69]. The goal of a SERM/CE pairing is to blend the positive effects of estrogens on menopausal symptoms and bone with the protective effects of a SERM on the endometrium and breast in women with a uterus [70]. The pairing of a SERM with CE may be an alternative to the use of progestins in EPT for treating non-hysterectomized, postmenopausal women.

#### Preclinical Evidence for Potential SERM/CE Combinations

The key issues a potential TSEC should address include endometrial and breast protection without counteracting the positive effects of estrogens on the central nervous system, skeleton, and vagina. Because each SERM and CE pairing will exhibit a different tissue selectivity profile, some pairings may not adequately achieve these goals. Results from preclinical studies evaluating various SERM/CE combinations further support this concept.

Preclinical studies assessing different SERM/CE combinations have shown promising results for the pairing of bazedoxifene (BZA) with CE, while data do not support the combination of CE with other SERMs. In mature/reproductively competent, ovariectomized (OVX) rats, coadministration of BZA with CE prevented CE-induced increases in uterine wet weight and preserved BMD [70,71]. Studies in OVX, sexually immature mice showed that BZA was more effective than raloxifene (RLX) and lasofoxifene (LAS) in inhibiting CE-induced increases in uterine wet weight [72]. In this model, BZA also demonstrated less agonist activity in the mammary gland and was a more effective antagonist of CE-induced breast stimulation than RLX and LAS [72]. Moreover, the addition of BZA did not inhibit the efficacy of CE in reducing tail skin temperature in a model of VMS [70].

Taken together, these preclinical data suggest that BZA/CE is a promising TSEC pairing, while the combination of RLX or LAS with CE may not be suitable, as these pairings cause unacceptable uterine stimulation. Published clinical studies of RLX combined with oral or transdermal estrogens further reinforce these preclinical findings and show endometrial stimulation with this SERM/estrogen combination [73,74,75]. In 2 clinical trials evaluating the combination of RLX and an oral estrogen (the first with 17β-estradiol [73] and the second with esterified CE) [75], both showed an increase in endometrial thickness from baseline after 52 weeks and 3 months of therapy, respectively. Another study reported a reduction in endometrial thickness with RLX plus PBO (−0.9 mm) compared with an increase in endometrial thickness with RLX plus low-dose transdermal estradiol after 8 weeks of therapy (0.8 mm; *p* = 0.021) [74]. In contrast, studies of other estrogen formulations (e.g., 17β-estradiol percutaneous or vaginal ring) administered in combination with RLX did not show signs of endometrial stimulation [76,77]. Based on these findings, BZA/CE was selected for further development and is the first TSEC in clinical development.

### 3.2. Clinical Studies of BZA/CE

The efficacy and safety of BZA/CE have been evaluated in non-hysterectomized, postmenopausal women in a series of randomized, double-blind, PBO- and active-controlled, phase 3 trials called the Selective estrogens, Menopause, And Response to Therapy (SMART) trials (Table 2). The SMART-1 trial (N = 3,397) was a 2-year study that evaluated the efficacy and safety of BZA/CE compared with RLX and PBO in women aged 40 to 75 years [78,79,80,81]. The primary endpoint was the incidence of endometrial hyperplasia. BZA 20 mg was shown in this study to be the lowest effective dose for protecting the endometrium from CE stimulation [81]. Based on these findings, BZA 20 mg/CE 0.45 and 0.625 mg were selected for evaluation in subsequent SMART trials. The 12-week SMART-2 trial (N = 318) evaluated BZA 20 mg/CE 0.45 and 0.625 mg compared with PBO in women aged 40 to 65 years who had ≥7 moderate-to-severe hot flushes daily at baseline [82,83]. The primary endpoint was the change from baseline in frequency and severity of hot flushes. The 12-week SMART-3 trial (N = 652) evaluated BZA 20 mg/CE 0.45 and 0.625 mg compared with BZA 20 mg and PBO in women aged 40 to 65 years with ≥1 moderate-to-severe VVA symptom [84,85]. The primary endpoint was the change from baseline in measures of VVA. Generally speaking, exclusion criteria for the SMART trials were based on the prescribing information for HT; therefore, criteria were similar for both the WHI and SMART trials. More specifically, relative to the WHI study population, women in the SMART trials were generally younger (WHI included women ages 50–79 years) and exclusion criteria were more restrictive in terms of cardiovascular risk factors, excluding women with a history or presence of thromboembolic disease, cerebrovascular event, or myocardial infarction/ischemic heart disease. In both the WHI and SMART trials, women with breast cancer were excluded from participation.

**Table 2:** Summary of the SMART trial study designs.

SMART-1 [78,79,80,81,86,87,88]	SMART-2 [82,83]	SMART-3 [84,85]
***Enrolled non-hysterectomized postmenopausal women***		
Aged 40–75 years with acceptable endometrial biopsy results at screening (N = 3,397)	Aged 40–65 years with acceptable endometrial biopsy results and ≥7 moderate-to-severe hot flushes/d at screening (N = 318)	Aged 40–65 years with acceptable endometrial biopsy results and ≥1 moderate-to-severe VVA symptom at screening (N = 652)
***Substudies***		
Osteoporosis substudy I: >5 years postmenopause with a baseline BMD T-score between −1 and −2.5 and ≥1 additional risk factor for osteoporosis (n = 1,454) Osteoporosis substudy II: 1–5 years postmenopause with ≥1 risk factor for osteoporosis (n = 861)	N/A	N/A
***Study duration***		
2 years	12 weeks	12 weeks
BZA 10, 20 and 40 mg/CE 0.45 and 0.625 mg RLX 60 mg PBO	BZA 20 mg/CE 0.45 and 0.625 mg PBO	BZA 20 mg/CE 0.45 and 0.625 mg BZA 20 mg PBO
***Primary endpoints***		
Incidence of endometrial hyperplasia at 1 year	Change from baseline in mean daily number and severity of hot flushes at Weeks 4 and 12	Change from baseline in proportion of vaginal superficial and parabasal cells, vaginal pH, and most bothersome VVA symptom at Weeks 4 and 12
***Secondary endpoints***		
Mean percent change from baseline in lumbar spine BMD at 2 years Mean percent change from baseline in total hip BMD Median percent change from baseline in serum BTM levels Change from baseline in mean daily number and severity of hot flushes Percent change from baseline in proportion of vaginal superficial, intermediate, and parabasal cells Incidence of breast pain Cumulative amenorrhea rates Percent change from baseline in breast density at 2 years (ancillary study) Evaluation of AEs	Sleep parameters (MOS sleep scale) QOL (MENQOL questionnaire) Treatment satisfaction (MS-TSQ) Incidence of breast pain Evaluation of AEs	Sexual function (ASEX scale) QOL (MENQOL questionnaire) Treatment satisfaction (MS-TSQ) Incidence of breast pain Evaluation of AEs

SMART, Selective estrogens, Menopause, And Response to Therapy; VVA, vulvar/vaginal atrophy; BMD, bone mineral density; BZA, bazedoxifene; CE, conjugated estrogens; RLX, raloxifene; PBO, placebo; BTM, bone turnover markers; AE, adverse events; MOS, Medical Outcomes Study; QOL, quality of life; MENQOL, Menopause-Specific Quality of Life; MS-TSQ, Menopause Symptoms-Treatment Satisfaction Questionnaire; ASEX, Arizona Sexual Experiences.

#### 3.2.1. Efficacy of BZA/CE on VMS

**Figure 1:**
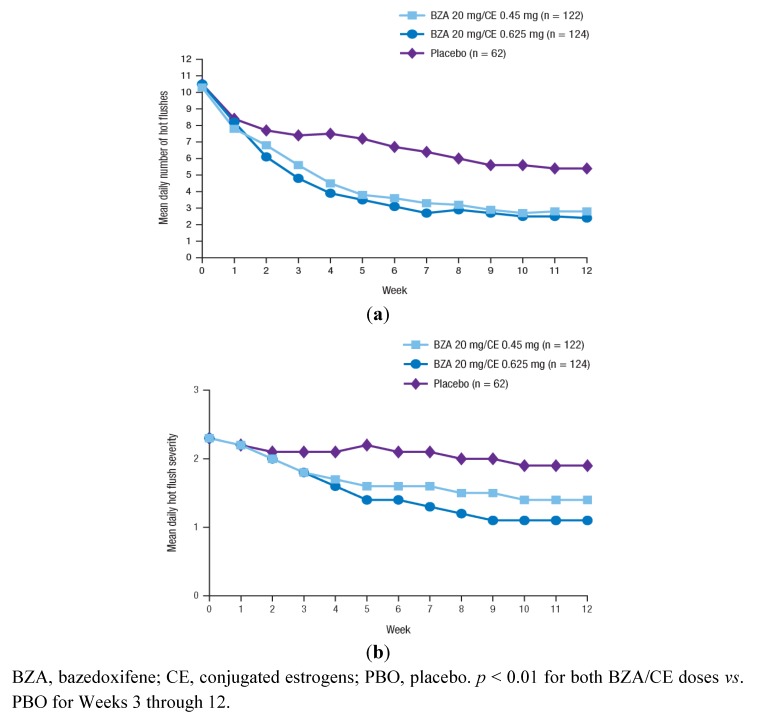
The mean daily number (**a**) and severity (**b**) of moderate-to-severe hot flushes over 12 weeks in the SMART-2 trial. Reprinted from Pinkerton *et al*. [82] with permission from Wolters Kluwer Health.

The effect of BZA/CE on VMS was evaluated in the SMART-1 trial in a subset of women with ≥7 moderate-to-severe hot flushes daily at baseline (n = 216) and in the SMART-2 trial (n = 310) [80,82]. In both studies, BZA 20 mg/CE 0.45 and 0.625 mg showed significantly greater decreases from baseline in the mean daily number and severity of hot flushes compared with PBO at 12 weeks (*p* < 0.05 for all; Figure 1 and Table 3). The efficacy of BZA/CE on VMS was sustained over 2 years of treatment in the SMART-1 trial [87]. In the SMART-2 trial, the efficacy of BZA/CE in reducing the frequency and severity of hot flushes compared with PBO was observed as early as Week 3; both BZA/CE doses also showed significantly higher percentages of subjects with at least a 50% or 75% decrease in the number of moderate-to-severe hot flushes at Weeks 4 and 12 compared with PBO (*p* < 0.001). Moreover, in a subanalysis of the SMART-2 study, the number of hot flush symptom-free days was significantly increased with both BZA/CE doses *vs*. PBO (*p* < 0.0001) over 12 weeks of therapy [89].

**Table 3:** Summary of key efficacy findings in the SMART trials.

SMART-1	SMART-2	SMART-3
***Efficacy on vasomotor symptoms***		
Significant reduction from baseline in the mean daily number of moderate-to-severe hot flushes at Week 12 for BZA 10, 20, and 40 mg/CE 0.45 and 0.625 mg *vs.* PBO (*p* < 0.05) [80] Significant reduction from baseline in the mean daily severity of hot flushes at Week 12 for BZA 10 and 20 mg/CE 0.45 and 0.625 mg *vs.* PBO (*p* < 0.001) [80]	Significant reduction from baseline in the mean daily number of moderate-to-severe hot flushes at Week 12 for BZA 20 mg/CE 0.45 and 0.625 mg *vs.* PBO (*p* < 0.01) [82] Significant reduction from baseline in the mean daily severity of hot flushes at Week 12 for BZA 20 mg/CE 0.45 and 0.625 mg *vs.* PBO (*p* < 0.001) [82]	N/A
***Efficacy on vulvar/vaginal atrophy***		
Significant increase from baseline in the proportion of superficial cells at 2 years for BZA 10 mg/CE 0.45 and 0.625 mg and BZA 20 mg/CE 0.625 mg *vs.* PBO (*p* < 0.01) [80] Significant increase from baseline in the proportion of intermediate cells and significant decrease in the proportion of parabasal cells at 2 years for BZA 10 and 20 mg/CE 0.45 and 0.625 mg *vs.* PBO (*p* < 0.001) [80]	N/A	Significant increase from baseline in the proportion of superficial and intermediate cells and significant decrease in the proportion of parabasal cells at 12 week for BZA 20 mg/CE 0.45 and 0.625 mg *vs.* PBO (*p* < 0.05) [85] Significant decrease from baseline in vaginal pH and improvement in the most bothersome VVA symptom at 12 week for BZA 20 mg/CE 0.625 mg *vs.* PBO (*p* < 0.05) [85]
***Effects on bone***		
Significant increase from baseline in lumbar spine BMD at 2 years for BZA 10, 20, and 40 mg/CE 0.45 and 0.625 mg *vs.* PBO (*p* < 0.001) [79] Significant increase from baseline in total hip BMD at 2 years for BZA 10, 20, and 40 mg/CE 0.45 and 0.625 mg *vs.* PBO (*p* < 0.05) [79] Significant reduction from baseline in serum BTM levels at 2 years for BZA 10, 20, and 40 mg/CE 0.45 and 0.625 mg *vs.* PBO (*p* < 0.001) [79]	N/A	N/A
***Effects on sleep and quality of life***		
N/A	Significant improvement from baseline in sleep parameters at 12 week for BZA 20 mg/CE 0.45 and 0.625 mg *vs.* PBO (*p* < 0.001) [83] Significant improvement from baseline in total and vasomotor function MENQOL scores at 12 week for BZA 20 mg/CE 0.45 and 0.625 mg *vs.* PBO (*p* < 0.001) [83]	Significant improvement in total, vasomotor function, and sexual function MENQOL scores at 12 week for BZA 20 mg/CE 0.45 and 0.625 mg *vs.* PBO (*p* < 0.05) [84]
***Satisfaction with treatment***		
N/A	Significantly greater treatment satisfaction at 12 week for BZA 20 mg/CE 0.45 and 0.625 mg *vs.* PBO (*p* < 0.05) [83]	Significantly greater treatment satisfaction at 12 week for BZA 20 mg/CE 0.45 and 0.625 mg *vs.* PBO (*p* < 0.05) [84]

SMART, Selective estrogens, Menopause, And Response to Therapy; BZA, bazedoxifene; CE, conjugated estrogens; PBO, placebo; N/A, not applicable; VVA, vulvar/vaginal atrophy; BMD, bone mineral density; BTM, bone turnover markers; MENQOL, Menopause-Specific Quality of Life.

Quality of life and patient satisfaction tools were also used to assess the efficacy of BZA/CE on VMS in the SMART-2 study. Significant improvements were reported with BZA 20 mg/CE 0.45 and 0.625 mg at 12 weeks compared with PBO in the vasomotor function domain of the MENQOL questionnaire and significantly greater satisfaction in the ability to control hot flushes during the day and night according to the Menopause Symptoms Treatment Satisfaction Questionnaire (MS-TSQ) [83]. A range of components of the Medical Outcomes Study (MOS) sleep scale also showed significant improvements with BZA/CE *vs.* PBO, including significant decreases in sleep disturbance (effect size [95% CI] *vs*. PBO: −0.65 [−0.98 to −0.31] for BZA 20 mg/CE 0.45 mg, −0.75 [−1.08 to −0.41] for BZA 20 mg/CE 0.625 mg) and significant increases in sleep adequacy (effect size [95% CI] *vs*. PBO: 0.55 [0.22 to 0.88] for BZA 20 mg/CE 0.45 mg, 0.59 [0.25 to 0.92] for BZA 20 mg/CE 0.625 mg) [83]. In summary, BZA/CE was associated with a clinically meaningful reduction in VMS, comparable to that observed with HT. Moreover, this efficacy was also correlated with improvements in sleep and menopause-related quality of life.

#### 3.2.2. Efficacy of BZA/CE on VVA

The efficacy of BZA/CE on measures of VVA was assessed in the SMART-1 trial in a subset of women with ≤5% superficial cells at screening who had a baseline and ≥1 on-therapy measurement (n = 1,867) [80]. In the SMART-3 trial, VVA parameters were evaluated in subjects who had a baseline and ≥1 on-therapy value for the parameter being assessed (n = 617, n = 637, and n = 634, respectively, for assessments of vaginal epithelial maturation, vaginal pH, and the most bothersome VVA symptom) [85]. Significantly greater increases from baseline in the mean proportion of superficial cells compared with PBO were observed for BZA 20 mg/CE 0.625 mg at 24 months in the SMART-1 trial and for BZA 20 mg/CE 0.45 and 0.625 mg at 12 weeks in the SMART-3 trial (*p* < 0.01 *vs*. PBO for all; Table 3). Both BZA/CE doses also showed significantly greater improvements from baseline in the mean proportions of intermediate and parabasal cells compared with PBO at 24 months and 12 weeks, respectively, in the SMART-1 and SMART-3 studies (*p* < 0.01 *vs*. PBO for all; Table 3). In the SMART-1 trial, the incidence of dyspareunia was significantly lower for subjects who received BZA 20 mg/CE 0.45 and 0.625 mg during Weeks 9 to 12 compared with those who received PBO (*p* < 0.05). In the SMART-2 trial, both BZA/CE doses showed a significantly greater improvement from baseline in vaginal dryness at Week 12 *vs.* PBO (*p* < 0.05). BZA 20 mg/CE 0.625 mg also showed a significantly greater decrease from baseline *vs.* PBO in vaginal pH (*p* < 0.001) and in the subjects’ most bothersome VVA symptom (*p* < 0.05) at Week 12. Taken together, these data support the ability of BZA/CE to improve vaginal health for non-hysterectomized postmenopausal women.

#### 3.2.3. Effects of BZA/CE on Bone

The SMART-1 trial included 2 osteoporosis substudies [79]. Substudy I (n = 1,454) enrolled women who had a BMD T-score between −1 and −2.5 at screening and ≥1 additional risk factor for osteoporosis, and whose last menstrual period (LMP) was >5 years prior to screening. Substudy II (n = 861) enrolled women with ≥1 risk factor for osteoporosis whose LMP was between 1 and 5 years prior to screening. In both substudies, all BZA/CE doses showed significantly greater increases from baseline in lumbar spine (*p* < 0.001) and total hip (*p* < 0.01) BMD at 12 and 24 months compared with PBO, which showed decreases from baseline (Table 3). The BZA/CE groups had significantly higher percentages of responders for lumbar spine BMD (defined as subjects who had no change or an increase from baseline in lumbar spine BMD at Months 12 and 24) compared with PBO in both substudies (*p* < 0.001). Substudy II also evaluated BTM, with all BZA/CE doses showing a significantly greater reduction from baseline in serum levels of osteocalcin and *C*-telopeptide (*p* < 0.001) compared with PBO at all time points assessed. These data for BZA/CE are supported by the established efficacy of each agent alone in osteoporosis. Single-agent BZA 20 and 40 mg/day significantly reduced the risk of new vertebral fractures compared with PBO in a large 3-year, phase 3 study; in a post hoc subgroup analysis of women at higher risk for fracture, BZA 20 mg was associated with a 50% reduction in nonvertebral fracture risk compared with PBO (*p* = 0.02) and of 44% compared with RLX 60 mg (*p* = 0.05) [90]. Likewise, as mentioned previously, ET showed significant reductions in total fracture risk during the WHI trial compared with PBO [27]. Taken together, these results suggest that BZA/CE may provide benefits in terms of fracture risk reduction in the postmenopausal osteoporosis population.

#### 3.2.4. Effects of BZA/CE on Sleep, Quality of Life, and Satisfaction with Treatment

BZA/CE was associated with improvement in quality of life and sleep. The effects of BZA/CE on sleep parameters were evaluated in the SMART-2 trial using the MOS sleep scale in randomized subjects who received ≥1 dose of study medication and had a baseline and ≥1 on-therapy measurement [83]. At Week 12, BZA 20 mg/CE 0.45 and 0.625 mg showed significant improvements in various sleep parameters including time to fall asleep, sleep disturbance, sleep adequacy, and sleep problems indexes I and II compared with PBO (*p* < 0.001; Table 3). BZA 20 mg/CE 0.625 mg also showed a significant improvement in sleep quantity *vs*. PBO (*p* = 0.01). Based on regression analysis, improvements in sleep parameters were significantly associated with reductions in the frequency of moderate-to-severe hot flushes.

Quality of life was assessed using the MENQOL questionnaire in the SMART-2 and SMART-3 trials in randomized subjects who received ≥1 dose of study medication and had a baseline and ≥1 on-therapy measurement [83,84]. In both trials, BZA 20 mg/CE 0.45 and 0.625 mg showed clinically significant improvements in vasomotor function and total MENQOL scores at 12 weeks compared with PBO (*p* ≤ 0.001; Table 3). Significant improvement in sexual function score was observed for BZA 20 mg/CE 0.625 mg in the SMART-2 trial and for both BZA/CE doses in the SMART-3 trial compared with PBO (*p* < 0.01 for all). BZA 20 mg/CE 0.625 mg also showed significant improvements *vs*. PBO in physical function score in both trials, and in psychosocial score in the SMART-2 trial (*p* < 0.0 for all). In the SMART-3 trial, sexual function was also evaluated using the Arizona Sexual Experiences (ASEX) scale. At Week 12, both BZA/CE doses showed significant improvement in ease of lubrication compared with PBO (*p* < 0.05).

In the SMART-2 and SMART-3 trials, satisfaction with treatment was evaluated using the MS-TSQ in subjects who received ≥1 dose of study medication and had ≥1 on-therapy measurement [83,84]. In both trials, significantly higher proportions of subjects who received BZA/CE reported overall satisfaction with treatment at Week 12 compared with those who received PBO (SMART-2 trial, 73.5% to 78.2% for BZA/CE *vs*. 44.4% for PBO; SMART-3 trial, 62.6% to 69.4% for BZA/CE *vs*. 47.4% for PBO; *p* < 0.05 for all; Table 3). BZA/CE-treated subjects reported significantly greater satisfaction than those who received PBO with the ability to control hot flushes during the day and night, the effect on quality of sleep, and the effect on mood or emotions (*p* < 0.05 for all).

#### 3.2.5. Safety and Tolerability of BZA/CE

BZA/CE treatment has been shown in the SMART trials to be generally safe and well tolerated in postmenopausal women with a uterus. Across the SMART-1, SMART-2, and SMART-3 trials, no significant differences were observed between the BZA/CE and PBO groups in the overall incidences of adverse events (AEs) or study discontinuations due to AEs (Table 4) [80,82,85,91].

##### 3.2.5.1. Cardiovascular

The incidences of cardiovascular AEs and VTEs were similar among the BZA/CE and PBO groups [80,82,85]. In the SMART-1 trial, cardiovascular AEs (including myocardial infarction, coronary artery disease, and coronary artery insufficiency) occurred in <1% and at a comparable incidence across treatment groups, and the relative risk of VTE for BZA/CE *vs.* placebo was 0.48 (95% CI, 0.05–4.66) [80].

##### 3.2.5.2. Endometrium

In the SMART-1 trial, BZA 20 mg/CE 0.45 and 0.625 mg showed low rates (<1%) of endometrial hyperplasia over 2 years of treatment, similar to those observed for PBO (Table 4) [81]. There were no significant differences between these BZA/CE doses and PBO in the mean change from baseline in endometrial thickness at 2 years. The incidence of endometrial polyps was generally similar with the BZA/CE groups compared with PBO (1.3%–1.6%) at all time points, with the exception of BZA 10 mg/CE 0.625 mg (6.25%) and BZA 20 mg/CE 0.45 mg (5.67%) at Month 24. Reports of ovarian cysts were distributed similarly among treatment groups. There were no cases of endometrial hyperplasia reported in the SMART-2 trial and the change from baseline in mean endometrial thickness at 12 weeks was similar among the BZA/CE and PBO groups [82]. The SMART-3 trial showed no increase in the incidence of endometrial disorders or ovarian cysts for BZA 20 mg/CE 0.45 and 0.625 mg compared with PBO [85].

##### 3.2.5.3. Breast

Based on the favorable breast safety observed with the individual components of BZA/CE [46,47,92,93,94,95], in theory, one would speculate that the combination would also exhibit a favorable breast safety profile. Indeed, breast safety findings for BZA/CE are consistent with these expectations. An ancillary study of the SMART-1 trial demonstrated that BZA 20 mg/CE 0.45 and 0.625 mg treatment did not increase mammographic breast density over two years of treatment compared with PBO (Table 4) [88]. Breast cancer risk in the SMART-1 trial was low and similar to that observed for PBO [96]. Consistent with this observation, a pooled analysis of data for BZA 20 mg/CE 0.45 mg and PBO from the SMART-1, SMART-2, and SMART-3 trials showed no differences between these groups in the incidences of breast-related AEs [91]. There were also no differences between the BZA/CE and PBO groups in the incidence of breast pain in the SMART-1, SMART-2, and SMART-3 trials [80,82,85].

**Table 4:** Summary of key safety findings in the SMART trials.

SMART-1	SMART-2	SMART-3
***Overall safety***		
Similar incidences of AEs among BZA/CE and PBO groups [80] No increase in VTE incidence for BZA/CE groups compared with PBO [80]	Similar incidences of AEs among BZA/CE and PBO groups [82] No VTEs reported in any group [82]	Similar incidences of AEs among BZA/CE and PBO groups [85]
***Endometrial safety***		
Low endometrial hyperplasia rates (<1%) at 2 years, similar to those for PBO, for BZA 20 and 40 mg/CE 0.45 and 0.625 mg [81] Minimal increases from baseline in endometrial thickness (<1 mm) at 2 years, similar to those for PBO, for BZA 20 and 40 mg/CE 0.45 and 0.625 mg [81]	No endometrial hyperplasia reported in any group [82] No difference in mean change from baseline in endometrial thickness at 12 week among the BZA 20-mg/CE 0.45- and 0.625-mg and PBO groups [82]	Similar incidence of endometrial disorders among the BZA 20-mg/CE 0.45- and 0.625-mg and PBO groups [85]
***Tolerability***		
High rates of cumulative amenorrhea over 2 years, similar to that for PBO, for BZA 20 and 40 mg/CE 0.45 and 0.625 mg [78] No difference in change from baseline in mammographic breast density at 2 years among the BZA 20-mg/CE 0.45- and 0.625-mg and PBO groups [88] No increase in breast pain incidence for BZA/CE groups *vs.* PBO [80]	No increase in breast pain incidence for BZA/CE groups *vs.* PBO [82]	No increase in breast pain incidence for BZA/CE groups *vs.* PBO [85]

SMART, Selective estrogens, Menopause, And Response to Therapy; AE, adverse event; BZA, bazedoxifene; CE, conjugated estrogens; PBO, placebo; VTE, venous thromboembolic event.

##### 3.2.5.4. Uterine Bleeding

BZA 20 mg/CE 0.45 and 0.625 mg were associated with high rates of cumulative amenorrhea over 2 years of treatment, similar to that observed with PBO [78,86]. A pooled analysis of data for BZA 20 mg/CE 0.45 mg and PBO from the SMART-1, SMART-2, and SMART-3 trials showed similar incidences of bleeding-related AEs between those groups [91].

## 4. TSEC Summary: Menopause Symptom Relief without a Progestin

Taken together, results from the phase 3 SMART clinical trial program indicate that BZA/CE has comparable efficacy to HT in terms of its beneficial effects on VMS, VVA, bone, sleep, and quality of life with an improved safety and tolerability profile, particularly in terms of a more favorable breast and bleeding profile. As mentioned earlier, data suggest that many of the tolerability concerns associated with HT appear to be related to the progestin component, particularly irregular bleeding [97,98] and breast pain/tenderness [16,60,99]. The favorable tolerability profile of BZA/CE relative to EPT (particularly CE/MPA) may be linked in part to the contrasting pharmacology of BZA *vs*. progestins. For example, irregular vaginal bleeding is a known treatment effect of EPT [97,98]; progestins cause changes in the endometrium associated with bleeding (*i.e.*, increased blood supply and angiogenesis). While these effects are considered a normal part of the menstrual cycle for premenopausal women, irregular bleeding and spotting are troublesome for the postmenopausal population and are a common reason for discontinuation of EPT [59,100]. The goal of a TSEC such as BZA/CE is to achieve an optimal blend of tissue-selective activities without the need for a progestin in women with a uterus, thus minimizing some of the tolerability concerns associated with this component. BZA in combination with estrogens, such as CE, acts as an estrogen receptor antagonist in the endometrium and on breast cancer cells [101,102]. These antagonist effects in the endometrium and breast may contribute at least in part to the favorable bleeding and breast profile observed with BZA/CE relative to that observed with EPT.

## 5. Conclusions

HT is the conventional and established therapy option for postmenopausal women, with EPT recommended for women with an intact uterus. Both ET and EPT have demonstrated efficacy in relieving VMS and symptoms associated with VVA, as well as in preventing osteoporosis. However, EPT, particularly CEE/MPA, may be associated with some safety and tolerability concerns. The appropriateness of HT should therefore be evaluated based on the benefits and risks for each individual woman. For non-hysterectomized women, there is a need for alternatives to EPT for treating menopausal symptoms and preventing osteoporosis while ensuring reproductive safety.

The TSEC provides a novel approach to treating menopausal symptoms and preventing osteoporosis while maintaining endometrial and breast safety through the pairing of a SERM with 1 or more estrogens. BZA 20 mg/CE 0.45 and 0.625 mg have been shown in the SMART trials to be effective in relieving VMS and VVA symptoms and preserving bone mass while protecting the endometrium and breast. In addition, these BZA/CE doses were associated with a favorable overall safety and tolerability profile over two years of treatment, with no increases in the incidences of VTEs and cardiovascular events compared with PBO. Consistent with findings from the SMART-1, SMART-2, and SMART-3 trials, the recently completed SMART-5 trial also demonstrated the positive effects of BZA 20 mg/CE 0.45 and 0.625 mg on bone, sleep parameters, and QOL [103,104], as well as its overall safety and tolerability in postmenopausal women [105,106]. Based on these findings, BZA/CE may be a promising alternative to conventional EPT for non-hysterectomized, postmenopausal women who are seeking a safe and comprehensive therapy for the relief of menopausal symptoms and the prevention of postmenopausal osteoporosis.
